# Impact on Carbon Intensity of Carbon Emission Trading—Evidence from a Pilot Program in 281 Cities in China

**DOI:** 10.3390/ijerph191912483

**Published:** 2022-09-30

**Authors:** Wanlin Yu, Jinlong Luo

**Affiliations:** School of Economics, Shandong University of Technology, Zibo 255000, China

**Keywords:** carbon emission trading pilot, carbon intensity, green technology innovation, environmental governance level

## Abstract

China’s carbon emissions trading scheme (ETS) is an institutional arrangement that China intends to explore as a means of energy conservation and emission reduction. It is the core of China’s goal of achieving carbon peaking and carbon neutrality. This paper regards the introduction of pilot carbon emission trading policies as a quasi-natural experiment. Propensity Score Matching (PSM), Differences-in-Differences (DID), and spatial Durbin methods were used to evaluate the policy effects of pilot carbon emission trading policies on the carbon intensity of Chinese cities. We empirically tested the impact mechanism using the panel data of 281 cities at the prefecture level and above in China from 2006 to 2019. The results show that (1) the pilot policy of carbon emission trading has significantly reduced the carbon intensity of Chinese cities and shows characteristics of heterogeneity; (2) the dynamic effect test shows that the mitigation effect of the pilot carbon emission trading policy has increased gradually with time; (3) the mediation effect shows that the pilot carbon emission trading policy alleviates urban pollution in the region by improving the level of environmental governance and jointly reduces urban carbon intensity by increasing the level of green technology innovation; (4) the Durbin test suggests that pilot carbon emissions trading policy enforcement can significantly improve the carbon intensity of the area surrounding the city. In summary, the national carbon emissions trading market appears to be a successful experiment that also can contribute to China’s sustainable development. Its promise in achieving the “double carbon” target provides important policy implications.

## 1. Introduction

In the context of economic globalization, climate change is a major challenge for the survival and development of mankind in the 21st century, while the economic development of countries around the world always comes at the cost of energy consumption [1]. The “Statistical Review of World Energy” released by BP shows that global energy demand grew 2.9 percent in 2018, while carbon emissions rose 2.0 percent to reach their highest point in the 21st century. Global primary energy consumption grew 2.9 percent, almost double the average growth rate of 1.5 percent over the past decade [2]. At the same time, carbon emissions from energy consumption grew by 2%, also the highest in years. The new carbon emissions amounted to 600 million tons, which is equivalent to adding a third of the emissions produced by the planet’s passenger cars. Therefore, it is of great significance to implement effective means to achieve rapid carbon peaking and net zero emissions [3].

As the world’s largest developing country, China has become the world’s largest carbon emitter. China’s carbon dioxide emissions reached 11.3 billion tons in 2021, accounting for 33 percent of the global total [4]. The Chinese government has announced its intentions to undertake increasingly forceful measures with the goal of achieving a carbon peak before 2030 and carbon neutrality by 2060 [5]. This demonstrates China’s determination to achieve its “dual carbon” goal of carbon emissions and carbon neutrality and to actively undertake the corresponding obligations of its international treaty obligations.

Carbon taxes and emissions trading systems are internationally recognized as effective tools to reduce carbon emissions. According to China’s current situation, in order to ensure people’s livelihoods, China temporarily does not tax carbon dioxide emitted by coal and natural gas used by individuals. For China, the ETS has become the main tool to reduce carbon emissions.

In December 1997, the Kyoto Protocol was adopted as the first additional agreement to the United Nations Framework Convention on Climate Change (UNFCCC). As part of that agreement, market mechanisms were recognized as a new path to reduce greenhouse gas emission—that is, the right to emit carbon dioxide became regarded as a commodity, thus forming the basis of carbon trading systems [6]. The European Emissions Trading System (EUETS), the world’s largest carbon market, came into operation in 2005. The scheme imposes emission limits on member countries; the sum of national emission allowances does not exceed the emissions allowed under the Protocol. The allocation of emission allowances takes into account factors such as historical emissions, projected emissions, and emission standards of member countries [7]. 

The EU emissions trading system uses “Cap-and-Trade” rules. In order to limit the total amount of greenhouse gas emissions, administrative permits for emissions are bought and sold. The three major principles are the total trade principle, decentralized governance mode, and development characteristics. Under the EUETS, EU member state governments must agree to national emission caps set by the EUETS. Within this cap, companies can sell or buy additional credits in addition to their allocated emissions, provided that overall emissions fall within a specific quota. Firms that emit excess emissions beyond their allocated or purchased allotment are penalized, while those with surplus allowances can keep the emissions for future use or sell them to other firms. The EUETS has played an exemplary role in the world’s development of carbon trading markets. 

China’s carbon market construction started with local pilots [8,9] based on the EUETS. In 2011, the Chinese government listed seven provinces and cities, including Beijing, as pilot areas of the ETS. In 2013, these pilot carbon markets began online transactions. The aim of the program is to cost-effectively reduce greenhouse gas emissions of enterprises in the pilot provinces and cities. The goals include training talent and accumulating experience to lay the foundation for a national carbon market [10]. At present, a national carbon market has started with the power generation industry (2225 enterprises). Eight industries with high energy consumption, including power, petrochemical, chemical, building materials, steel, non-ferrous, paper-making, and civil aviation, will be included in the national carbon market. It is expected to gradually include another seven industries over the 14th Five-Year Plan period.

The carbon emission trading scheme (ETS), regarded as a vital market-driven carbon mitigation instrument, could trigger technology innovation and accelerate a green economic transition [11]. In 2015, China’s CO_2_ emission from fossil fuel consumption was about 9 billion tons. During the 14th Five-Year Plan period, overall carbon intensity is expected to decrease by 18% and energy consumption per unit of GDP will be reduced by 13.5 percent. Now, the ETS has introduced a system innovation. How to reduce the carbon intensity of cities? What are the pathways that affect carbon intensity? This study will evaluate the ETS policy from the perspective of regional carbon emissions. A thorough review of the pilot policy’s impact on carbon emissions, and its relationship to China’s overall development, will provide valuable experience for China’s efforts to deepen the reform and transformation of its pattern of economic development.

The rest of this study will be divided into the following parts. Part 2 is a literature review. Part 3 is a theoretical hypothesis. Part 4 is the data and empirical framework. Part 5 is the regression analysis. Part 6 further analyzes the mediating effect and spillover effect. Part 7 concludes and makes policy recommendations.

## 2. Literature Review

Because carbon emissions cause negative externalities [12], the arguments of Pigou [13] suggest government intervention through means such as taxation. Coase [14] held the opposite opinion, believing that the government should regulate property rights and allow the market to respond to externalities. In both cases, the instruments of the market are used to address externalities. Dales [15] proposed commercializing pollution on the basis of Coase, arguing that the pollution caused by companies is the property of the government, and that businesses should be able to buy and sell freely in the market. This was the embryonic form of the modern emissions trading system. 

As mentioned above, although China’s ETS has borrowed some practices from the EUETS, it is different. First, the EUETS consists of a “three-pillar” system of “carbon trading”, “carbon tax”, and “carbon border tax”. This is slightly different from a carbon emission quota, which is the basis of carbon emission trading in China. Second, the EU emissions trading scheme adopted a cap-and-trade principle. That is, on the premise that the total amount of emissions does not exceed the allowable upper limit, each emission source can adjust its emissions through exchange of permits. The upper limit will be reduced year by year. By contrast, carbon trading in China is divided into a primary market and a secondary market. The primary market is mainly for “quota creation”, which is managed by national authorities and entrusted to agencies to create and distribute carbon emission rights quotas. The participants in the secondary market are mainly enterprises and financial institutions. Third, the trading rules published by the Shanghai Ring Exchange have price fluctuation limits within a daily limit. The EU carbon price, on the other hand, has no price limit. Carbon prices in the European Union have risen rapidly in recent years, more than doubling from pre-pandemic levels. Fourth, the industry coverage of the EU carbon trading system, which started with the power industry and energy-intensive industries, gradually expanded to the transportation sector and the production of specific products such as steel and cement. At present, China’s carbon emission trading market is focused on the electric power industry [16,17].

Existing research on emissions trading can be broadly divided into two categories. The first category focuses on assessing the efficiency of the ETS design, including the effectiveness of a carbon price in reducing emissions [18,19], the controllability of transaction costs [20,21], and the rationality of quota allocation [22,23]. The second category focuses on how the ETS affects macroeconomic variables. This study is in the second category.

From the perspective of energy conservation and emission reduction, earlier studies mostly used scenario simulation to evaluate carbon emission trading. In terms of energy saving, most scholars have used data simulation analysis. It has been found that ETS can effectively reduce the consumption of non-renewable energy [24,25]. In terms of emission reduction, Zhang et al. [26] simulated ETS implementation in China and found that inter-regional commodity exchanges can alleviate carbon emissions, based on China’s provincial panel data [27]. The simulations were analyzed in the case of both unconstrained and constrained countries to assess the potential effectiveness of ETS in China. The study found that ETS had the potential to reduce carbon intensity by 20.06% without having a negative effect on GDP.

The development of the ETS systems in Europe and China provides the opportunity to turn the simulation into reality. Most studies have found that ETS has reduced carbon in pilot areas in China. Computable General Equilibrium (CGE) and Difference in Difference (DID) models have been the main empirical evaluation methods used in recent years. Liu et al. [1], using a regional CGE model, found that the Hubei province pilot ETS reduced carbon emissions by about 1% in 2014. In an empirical study, Yucai et al. [28] used DID to model the effect of the pilot ETS on energy conservation and emissions reduction; the results showed that regulated industry energy consumption in the ETS pilot areas decreased by 22.8% and carbon emissions by 15.5%.

Some scholars also have studied the possible economic losses caused by the implementation of ETS. Most scholars have found that EUETS has had no adverse effect on corporate profits and social welfare [29]. For China’s carbon trading market, however, Wang and Pan [30] found that the implementation of ETS has led to a 0.28% decline in GDP. This is because China’s economic development has been dependent on natural resources. Hubler et al. [31] found that the economic losses of ETS in China may be around 1%.

In conclusion, the existing papers mainly study the impact of ETS on energy saving, emission reduction, and economic loss. However, there are few studies on comprehensive macro indicators, such as urban carbon emission intensity. Urban carbon emission intensity is defined as the ratio of CO_2_ emissions to GDP in a city within a year. This indicator has been widely used to evaluate China’s “double carbon” target [32].

In China, most studies on ETS use a CGE model or a DID model. There are a number of limitations with these studies. CGE modeling is subject to defects such as difficulty in meeting the assumptions on which it is premised, strong subjectivity of parameter setting, and difficulty in determining whether its feedback mechanism measures real effects. The DID model requires homogeneity of the sample, whereas in reality, there is heterogeneity in relevant characteristics between the treated and control localities. In addition, most of the relevant studies start from the provincial level, while implementing carbon emission trading policies depends more on whether urban units can strictly implement the orders of their superiors. Further, earlier studies have ignored the influence of spatial factors on carbon intensity, although spatial factors have an important impact on carbon intensity and neglecting spatial factors may lead to bias in simulation results.

Against this background, this study makes the following contributions. First, the introduction of pilot carbon-emission trading policies is regarded as a quasi-natural experiment. This allows the use of a PSM-DID model estimation method to assess the impact of ETS on urban carbon intensity. The quasi-natural experiment not only meets the requirements of a DID model, but also ensures optimal matching because of the large samples. This gives more credibility to the research conclusions. Second, this study focuses on carbon intensity at the city level. Considering that cities are an important part of local government institutions in China, this makes the policy effect more plausible. Third, this study uses spatial Durbin to test the spillover effect of ETS on surrounding areas, thus going beyond the previous focus on the local area, which has ignored the surrounding area. This provides a more complete picture of the impact of emissions trading policies.

## 3. Theoretical Background

The carbon emission trading system is mainly an exercise of the “Porter hypothesis,” which holds that appropriate environmental regulation can encourage enterprises to carry out more innovative activities [2]. These innovations will increase the productivity of firms, thereby offsetting the costs of environmental protection and reducing total carbon emissions at the societal level. Theoretically, the system is dominated by the government, which uses market mechanisms to promote energy conservation and emission reduction [33]. First, the ETS sets a relatively strict carbon allowance for each company. Within this limit, companies can carry out free carbon emissions. The excess needs to be purchased from the carbon emissions retained by other companies. In essence, carbon permits have become a commodity [34]. Because firms aim at profit maximization, companies make good use of free credits while trying to avoid exceeding that limit; otherwise, high production costs will be incurred. Second, ETS can promote corporate emission reduction because firms will sell unused emissions credits if the carbon price is higher than the firm’s marginal cost of emission reduction [35]. Therefore, a market-based trading system can be effective in mitigating carbon emissions.

Establishing a carbon emission trading system can force enterprises to innovate, and technological progress is one of the three major factors affecting the environment [36]. Green technology innovation depends on increasing investment in such innovation. The emissions trading system encourages companies to actively develop and apply green technologies [2]. Companies that invest more resources in reducing carbon emissions can sell surplus carbon emission credits to high-carbon emission enterprises and obtain high profits [37]. Firms will tend to accelerate the process of green technology development in order to achieve higher profits. This is the incentive effect. Conversely, for high carbon emission enterprises, it is necessary to buy carbon emission credits from sellers, which will increase production costs, compress profit margins, and reduce these firms’ competitiveness. Under this pressure, enterprises have to carry out technological innovation [2]. This is the punishment effect.

The incentive effect and punishment effect of market-based environmental regulation such as ETS give the government more tools for environmental governance. Because carbon dioxide does not harm health or production in the short run, and because it is costly to enforce non-market forms of governance, it has been difficult for the focus of environmental governance to shift quickly in the direction of reducing carbon emissions. By encouraging innovation and providing opportunities for profit, ETS has effectively improved the environmental governance level while ensuring that normal activities and production can continue. 

**Figure 1:**
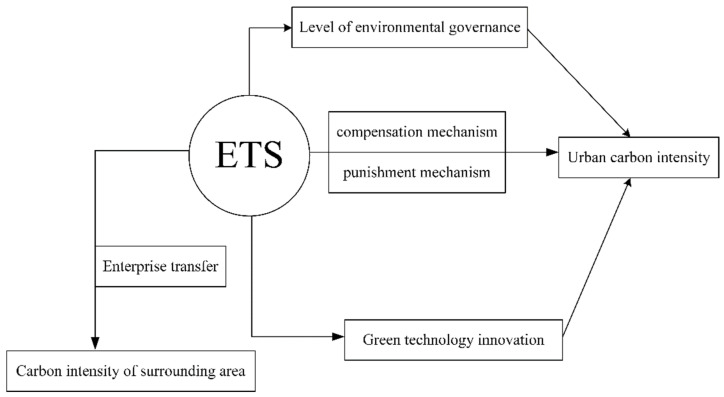
Theoretical background of the study.

Improved environmental governance can promote a change of regional energy structure. In particular, ETS has the potential to reduce coal consumption [28]. This paper applies the new economic geography to evaluate such changes. Firms will always look for the optimal location in order to maximize profits [38]. Theoretically, a carbon emission trading system should have policy spillover effects [39], including alleviating regional carbon emissions. It can also encourage high-tech enterprises to continue to innovate through its incentive mechanism. However, it will also cause a large number of polluting enterprises to incur high production costs due to its punishment mechanism. This is because polluting enterprises in the region face increased production costs due to the need to buy carbon emission rights, which reduces their profits. If there is no ETS policy in the surrounding areas, polluting enterprises are expected to migrate to the surrounding areas. Conversely, high-tech firms from surrounding areas are expected to migrate to the ETS area in order to increase their profits by selling carbon credits. The transfer behavior of the two types of enterprises can reduce carbon emissions in the ETS region while increasing emissions in the area around the ETS [40]. The theoretical background of this study is shown in Figure 1. Accordingly, the following hypothesis is proposed:

**Hypothesis** **1** **(H1).** *The carbon emissions trading system has reduced the carbon intensity of the pilot cities in China*.

**Hypothesis** **2** **(H2).** *Green technology innovation is one mechanism through which the carbon emissions trading system reduces regional carbon intensity*.

**Hypothesis** **3** **(H3).** *Improving urban environmental governance is another mechanism through which the carbon emission trading system alleviates regional carbon intensity*.

**Hypothesis** **4** **(H4).** *The carbon emission trading system has increased the carbon intensity of the areas surrounding the pilot cities*.

## 4. Data and Methodology

### 4.1. Data Sources

Through screening and matching, this paper selected panel data of 281 cities in China from 2006 to 2019 as the research object. A total of 37 cities at the prefecture level and above were designated as pilot carbon emission trading cities. These 37 cities constitute the experimental group, and the remaining cities were analyzed as the control group. Most of the data in this study come from data already publicly available in China, including the National Bureau of Statistics (https://data.stats.gov.cn/, accessed on 15 August 2021), the China City Statistical Yearbook, the China Energy Statistical Yearbook, and the Statistical Yearbooks of 281 cities. The data were drawn from reports on social development, including statistics on the main energy consumption of local industries above city size, total industrial output value, urbanization rate, etc. Patent data were derived from the State Intellectual Property Office (https://www.cnipa.gov.cn/, accessed on 20 August 2021).

### 4.2. Variable Selection

Urban carbon intensity is based on the total amount of carbon emissions to be measured. In this study, a material balance algorithm was used to calculate the total carbon emissions. Carbon emissions are estimated using the chemistry of carbon dioxide produced during energy consumption.

(1) $$Carbon_{it}=\sum_{v=1}^{n}Q_{vt}\times W_{v}\times M_{v}\times R_{v}\times 44/12$$

$Q_{vt}$ is the annual actual consumption of the V type of energy in the city in year t. According to the 26 fossil fuels listed in the China Energy Statistical Yearbook, they are combined into nine final energy consumption types: coal, coke, crude oil, gasoline, kerosene, diesel, fuel oil, natural gas, and electricity. Because electricity is not a direct energy source, the concept of secondary energy reflects the fact that electricity is produced by consuming other energy; therefore, this study will not measure electricity separately. $W_{v}$, $M_{v}$, $R_{v}$ are the energy calorific value conversion coefficient, carbon emission coefficient, and carbon oxidation factor, respectively. The data come from the average low calorific value of the China Energy Statistical Yearbook and IPCC (2006). As (44/12) is known to be the ratio of carbon dioxide to carbon molecular weight, the carbon dioxide emissions of 281 cities in China from 2006 to 2019 can be calculated. Since this study uses historical CO_2_ emission data, and is not based on carbon trading schemes, it should be considered post hoc analysis, and therefore calculation errors caused by different ways of allocating emission reduction targets can be avoided.

By referring to relevant literature and considering the actual situation [34], the following control variables were selected to conduct propensity matching scores: regional economic development level (PGDP), industrial structure (IND), urban population (PP), degree of openness to the outside world (OPEN), efficiency of financial development (FS), scale of financial development (FD), and government environmental intervention (WODK). The specific calculation methods are shown in Table 1.

### 4.3. Model Setting

The reference measurement model of this paper is set as follows:

(2) $$Carbon_{it}=\alpha_{0}+\alpha_{1}treated_{it}*time_{it}+\sum_{i=1}^{N}\beta_{j}control_{it}+\mu_{i}+\gamma_{t}+\varepsilon_{it\;}\;$$

where i represents the individual city and *t* represents the year. $Carbon_{it}$ is the carbon emission of city i in year t. The year dummy variable $\;time_{it}$ takes a value of 0 before the introduction of the carbon emission trading pilot policy (the policy impact point is set as 2013) and 1 after the establishment, and $treated_{it}$ is a group dummy variable. The ETS pilot cities are assigned a value of 1, non-ETS pilot cities are assigned a value of 0, and $treated_{it}*time_{it}$ is the interaction term of the two and takes the value of 0 or 1. Here, 1 represents the pilot cities after 2013, and 0 represents non-pilot cities and the pilot cities before 2013. The coefficient $\alpha_{1}\;$ before the interaction term of $treated_{it}*time_{it}$ is an important explanatory variable that represents the policy effect of emissions trading on urban carbon intensity. This paper will introduce various control variables affecting urban carbon intensity into the multi-stage DID regression. The bidirectional fixed effect of city and year will be introduced for further analysis.

### 4.4. Propensity Score Matching Results and Descriptive Statistics

#### 4.4.1. Counterfactual Matches with the Equation Estimates

Rosembaum and Rubin proposed the Propensity Score Method [41]. Simulation experiments show that the ATT can obtain unbiased estimation results under a series of assumptions. It can be defined as “an algorithm that matches the treatment group and the control group based on the conditional probability of participants, namely the propensity score, under the condition of given observable characteristics”. The propensity score is defined as:

(3) $$P\left(X_{i}\right)=Pr\left\{exp_{i}=1|X_{i}\right\}$$

According to Equation (3), the propensity score similarity between the treatment group and the control group is matched, and its effectiveness depends on two preconditions. The first is conditional independence. The second is that the conditions for common support are met. The independence condition means that ETS pilot cities or non-pilot cities are independent of carbon intensity after controlling the common influencing factor X, and the common support condition ensures that cities in each treatment group can match cities in the control group through propensity score matching. The average treatment effect ATE of city *i* can be expressed as

(4) $$E\left[{△}_{i}\right]=E\left[lny_{i}^{1}\left(ny_{i}^{1},fy_{i}^{1}\right)|exp_{i}=1,P\left(X_{i}\right)\right]-E\left[lny_{i}^{0}\left(ny_{i}^{0},fy_{i}^{0}\right)|exp_{i}=1,P\left(X_{i}\right)\right]$$

To estimate *P(X)* is to estimate the probability that the city is or is not an ETS pilot. A Probit or Logit binary choice model is most commonly used. In this paper, a Probit model is used to obtain the predicted probability value *P_i_* of city *i* in the treatment group and *P_j_* of city *j* in the control group. The average treatment effect (ATT) of ETS on carbon intensity is as follows:

(5) $$\beta =\frac{1}{M}\sum_{i\in \left(exp=1\right)}\left[lny_{i}\left(ny_{i},fy_{i}\right)-\sum_{i\in \left(exp=0\right)}Y\left(NY,FY\right)\left(p_{i},p_{j}\right)lny_{j}\left(ny_{j},fy_{j}\right)\right]$$

where *M* is the number of cities in which ETS was piloted. $Y\left(NY,FY\right)\left(p_{i},p_{j}\right)$ represents the case when $lny_{i}^{0}\left(ny_{j}^{0},fy_{j}^{0}\right)$ of city j is replaced by $lny_{i}^{0}\left(ny_{j}^{0},fy_{j}^{0}\right)$ of city *i*. This represents the weight assigned to $lny_{i}^{0}\left(ny_{j}^{0},fy_{j}^{0}\right)\;$ of city *j*. When the corresponding assumptions are met, especially when the mean values of variables in the treatment group and the control group are not different, the propensity score matching method can obtain the ATT, and a “clean” policy treatment effect can be obtained. Of course, being able to eliminate this noise completely requires being able to control for all variables that may have an impact on choice and outcome when matched. According to the matching method (radius matching, caliper matching, local linear regression matching, etc.), the weight function selection is also different. This study first selects the nearest neighbor matching with k = 4, and then selects other matching methods in the robustness test.

#### 4.4.2. Plot of Propensity Score Matching Kernel Density Function

**Figure 2:**
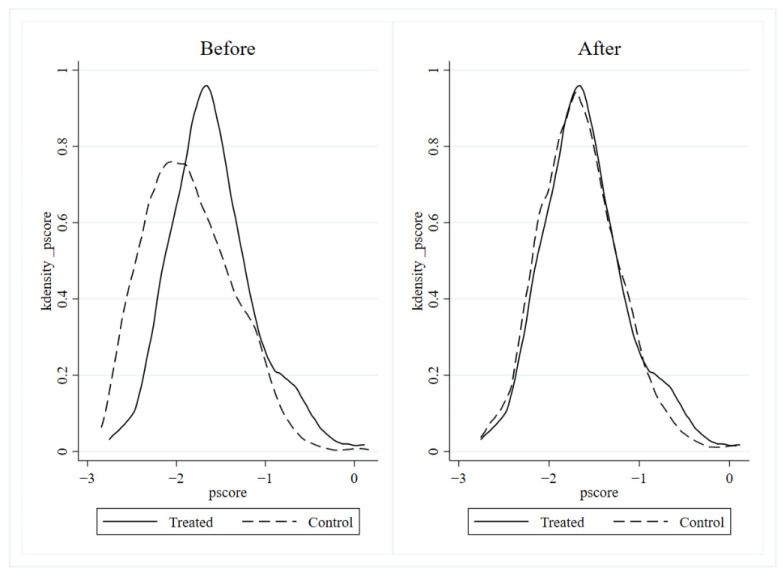
Kernel density function before and after PSM in treatment group and control group.

The quality of propensity score matching can be examined by a plot of kernel density functions. If there is more overlap between the treatment group and the control group in the figure, this indicates that the test propensity match score is better. Figure 2 shows the kernel density function of the two groups of cities before and after propensity score matching. The solid line represents the cities in the processing group, and the dashed line represents the cities in the control group. As shown in Figure 2, prior to PSM, the two groups showed large differences in both skewness and kurtosis. After PSM, the change trend of the two groups is consistent, and there is a high degree of line segment coincidence. This indicates that the propensity score matching has a significant effect. This provides a good data basis for the use of the DID method in the empirical part of this paper. 

#### 4.4.3. Balance Test of Propensity Score Matching and Variables of Descriptive Statistics

In order to make the results of PSM more robust, the results should satisfy the two groups of cities, and there is no obvious difference in each matching variable. The method to judge whether PSM is effective generally carries out the balance test of propensity score matching. Note the absolute value of the standard deviation of the matching variable. If the absolute value of the standard deviation is smaller, it indicates that the matching effect is better. Table 2 results show that most of the matching variables decrease significantly in the absolute value of the PSM standard deviation. The t-test value also changed from significant to insignificant. This indicates that the null hypothesis that the mean of each variable is consistent after matching is accepted. Propensity matching scores are valid. Table 3 shows the descriptive statistics of variables after PSM.

## 5. Empirical Analysis

### 5.1. Results of Dual Difference Regression

In order to more clearly identify the causal impact of ETS on urban carbon intensity, the above control variables will be introduced in this section. The model combining city individual fixed effects (Id) and year fixed effects (Year) is used for further analysis, and the results are shown in Table 4. The results in column (1) show that, without adding any control variables, the coefficient of Treated*time is significantly negative at the level of 1%. The results in columns (2)–(4) show that, after the introduction of other control variables, the coefficient of Treated ∗ time is significantly negative at the 1% level. This indicates that ETS can effectively reduce urban carbon intensity. In order to make the results more reliable, Column (5) shows the test results of the generalized method of moments estimation for dynamic instrumental variables. The coefficient of Treated ∗ time is still significantly negative at the level of 1%, which further verifies the conclusion of this paper. This empirical study also preliminarily shows that the introduction of pilot carbon emission trading policies can effectively reduce urban carbon intensity, and Hypothesis 1 is established.

### 5.2. Heterogeneity Analysis

#### 5.2.1. Regional Heterogeneity Test

On the whole, ETS can effectively reduce the carbon intensity of cities. However, different cities in China are located in different external environments. This leads to obvious differences across urban regions. In particular, the pilot policy of carbon emission trading has great relevance to the energy environment. The economically developed eastern region and the economically less-developed central and western regions have obvious differences in infrastructure and other conditions. Table 5 shows the regional heterogeneity results. The results in columns (1)–(3) show that the eastern region is inferior to the central and western regions in terms of coefficient and significance level. This indicates that the ETS policy in the eastern region is less effective in reducing carbon intensity. Relative to the central and western regions, the eastern region has a large population and more developed economy, with a concentration of various industries. As a result, the consumption of electric energy and heat energy caused by industrial electricity and residential electricity is large. Due to the normal economic and social activities in the eastern region, ETS cannot reduce the carbon intensity of the city in a short time. However, in China’s central and western areas, the population is lower and the economic development level is weaker. In those regions, the establishment of carbon emission trading pilots can effectively reduce the carbon intensity.

#### 5.2.2. Quantile Regression Test

**Figure 3:**
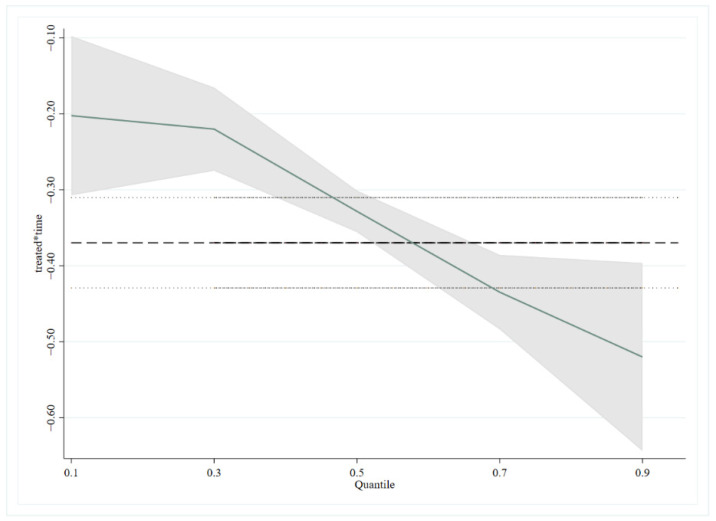
Quantile regression trend of carbon intensity of cities under ETS.

The regional heterogeneity of ETS on urban carbon intensity has been analyzed above. This part will analyze the quantile heterogeneity of ETS on carbon intensity—that is, the policy effect of ETS on high and low carbon intensity. It can be seen from Table 6 that, regardless of the value of M (M is the quantile), ETS always has a dampening effect on carbon intensity. Moreover, the impact of ETS on carbon intensity at different quantiles also changes significantly. Specifically, the emission reduction effect of ETS on cities with higher carbon intensity is more obvious. Figure 3 shows the trend of ETS regression on urban carbon intensity quantiles. The horizontal axis in the figure shows the different quantile decimal points of the ETS on urban carbon intensity. The vertical axis shows the regression coefficients of each variable. The dashed lines of the line segments represent the OLS regression estimates of the corresponding explanatory variables. The region between the two dotted lines represents the confidence interval of the OLS regression value (confidence 0.95). The solid lines are the quantile regression estimation results of each explanatory variable. The shaded part is the confidence interval (confidence 0.95) of the quantile regression estimate. Figure 3 further shows that the emission reduction effect of ETS on cities with higher carbon intensity is more obvious.

### 5.3. Robustness Test

#### 5.3.1. Parallel Trend Test

This section presents the results of parallel trend tests. The specific test formula is set as:

(6) $$Carbon_{it}=\alpha_{0}+\omega_{d}\sum_{d=-5}^{d=5}treated_{it}*time_{it}+\sum_{i=1}^{N}\beta_{j}control_{it}{\;}_{\;}+\mu_{i}+\gamma_{t}+\varepsilon_{it}\;$$

**Figure 4:**
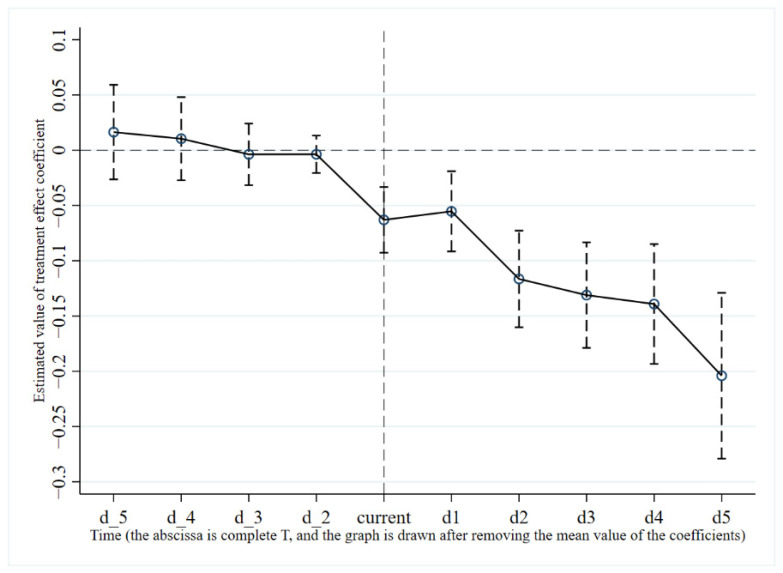
Parallel trend test.

The main variables in the above formula have the same meaning as in Formula (1), where d_5 represents 5 years before the introduction of ETS policy, and d5 represents the 5th year after the introduction of ETS policy. The coefficient $\;\omega_{d}$ is the focus of this paper’s test. If the coefficient estimate is insignificant before ETS, significantly negative after ETS, and shows a difference in marginal effects, then the parallel trend assumption is satisfied. As shown in Figure 4, before the ETS, the effect on carbon intensity is not significant. After the establishment of the ETS, the coefficient is significantly negative. The marginal effect of ETS on carbon intensity mitigation is strengthened over time, showing a long-term emission reduction effect. This proves the rationality of using the PSM-DID method in this paper. 

#### 5.3.2. Change the Sample-Matching Method

The nearest neighbor matching method with K = 4 was selected above for data matching and processing. In order to make the above conclusion more robust, this part re-selects the matching party for data matching. In this part, the methods of Mahalanobis distance matching, caliper matching, radius matching, and kernel matching were used to re-match the data. Table 7 shows the results of difference-in-differences estimation by various matching methods. After changing the propensity matching scoring method, the estimated results are close to the regression results above. This indicates that the above regression results are reliable, verifying that that ETS can effectively reduce urban carbon intensity.

#### 5.3.3. Placebo Test

**Figure 5:**
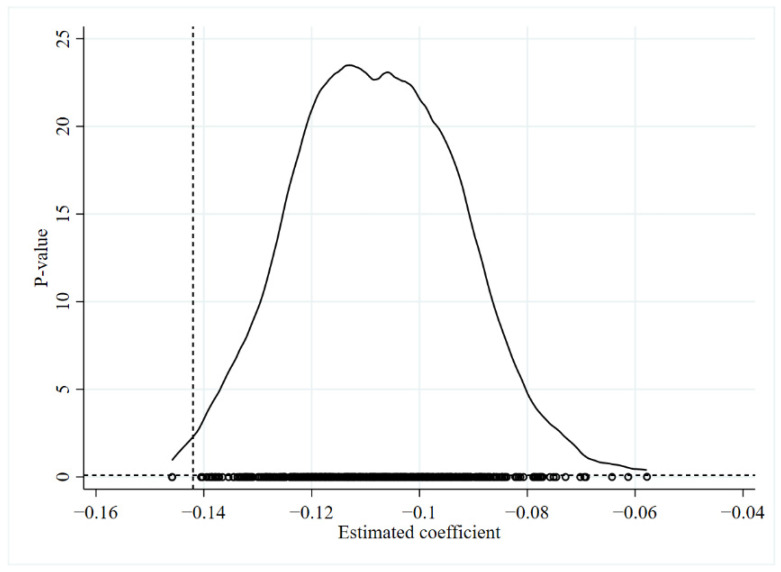
Placebo test.

The cities in which ETS was piloted may have been chosen as pilots due to their relatively complete infrastructure and high economic development potential. Therefore, in order to eliminate the interference of other unobservable factors with the conclusions of this paper, a placebo test was used to further prove the reliability of the previous conclusions. In this part, the interaction terms are randomly selected 1000 times to check whether the coefficients are significantly different from the benchmark estimation results. The results are shown in Figure 5. The dashed line indicates that the actual estimated coefficient obtained by PSM-DID is −0.142. The coefficient estimate is lower than 1000 random draws. This indicates that the placebo test in this part is valid. Thus, the reliability of the conclusions of this paper is proven.

## 6. Further Analysis

### 6.1. The Mediation Effect Test

The above empirical results show that introducing the carbon emission trading pilot policy has alleviated the carbon intensity of cities. Then, how does ETS affect the carbon intensity, and what is the specific mechanism? According to the above theoretical analysis, this paper argues that the pilot carbon emissions trading policy acts through green technology innovation and environmental governance. Therefore, this paper will examine the intermediary mechanism from the two channels of green technology innovation and environmental governance.

(7) $$M_{it}=\beta_{0}+\delta_{2}treated_{it}*time_{it}+\sum_{i=1}^{N}w_{j}control_{it}+\mu_{i}+\gamma_{t}+\varepsilon_{1it}\;$$

(8) $$Carbon_{it}=\theta_{1}+\delta_{3}treated_{it}*time_{it}+\delta_{4}M_{it}+\sum_{i=1}^{N}e_{j}control_{it}+\mu_{i}+\gamma_{t}+\varepsilon_{2it}\;$$

In the above equation, M represents the mediating variables, which are green technological innovation (Inno) and environmental governance (Trash), respectively. Among them, green technological innovation is represented by the number of green invention patents and green utility model patents granted per capita in cities [42]. A larger value indicates a higher level of green technology innovation. The calculation of the environmental governance level index is measured by the sum of waste water, waste gas, and solid waste generated by the city. A smaller value indicates a higher level of environmental governance.

Traditional parameter estimation methods require the assumption of a normal distribution of data. The use of stepwise regression may have some impact on the assessed policy effects. Therefore, the Sobel test and Bootstrap method were used to test the mediating effect in this part. The Bootstrap test uses the mixed effects hypothesis. In this paper, the original sample was randomly sampled repeatedly with *n* = 1000. The asymmetry in the distribution of indicators was corrected. This can significantly improve the accuracy of model testing under a complex mediation structure.

Table 8 shows the mediation test results. When Inno is used as a mediating variable, the coefficient before Treated ∗ time is significantly positive at the 1% level. This indicates that the introduction of the pilot policy of carbon emission trading has promoted the level of urban green technological innovation. The coefficient of urban carbon intensity is significantly negative at the 1% level. This shows that the improvement of green technology innovation alleviates urban carbon intensity, and the path of “carbon emission trading pilot policy-green technology innovation-urban carbon intensity” is established. This proves Hypothesis 2. 

When the level of environmental governance is used as a mediating variable, the coefficient before Treated ∗ time is significantly negative at the 1% level. This shows that the introduction of the pilot policy of carbon emission trading has improved the level of urban environmental governance. The coefficient of urban carbon intensity is significantly positive at the 1% level. This indicates that the improvement of environmental governance will alleviate urban carbon emissions. The path of “Carbon emission trading pilot policy—environmental governance level—urban carbon intensity” is established. This proves Hypothesis 3.

### 6.2. Spatial Spillover Effect Test

#### 6.2.1. Model Set and Related Analysis

According to the above analysis, the impact of the pilot carbon emission trading policy on carbon intensity may have a spatial spillover effect, which needs further analysis. Therefore, this paper establishes a spatial econometric model based on the equation:

(9) $$Carbon_{it}=\beta_{0}+\beta_{1}W\times Carbon_{it}+\beta_{2}CD_{it}+\beta_{3}W\times CD_{it}+\sum_{i=1}^{N}q_{j}control_{it}+\mu_{i}+\gamma_{t}+\varepsilon_{it}\;$$

where *W* is the spatial weight matrix. Equation (7) adds the spatial interaction term (*W* × *CD*) of the core explanatory variable (*CD*) to the equation. The model estimates the spatial spillover effects of the explained and core explanatory variables. Regarding the selection of the spatial weight matrix, this paper chooses the geographical inverse distance matrix to study the possible spatial spillover effect.

Before the spatial econometric analysis, it is necessary to determine whether there is a spatial correlation of urban carbon intensity. In this paper, the global Moran’s I index is used to test the spatial correlation of carbon emissions. Table 9 reports the regression results of each year. For 2006~2019, Moran’s I index shows significance under the 1% level, which shows a spatial correlation in urban carbon intensity.

#### 6.2.2. Analysis of Regression Results

Table 10 shows the regression results of the spatial Durbin model with double fixed effects. Columns (1)–(3) represent the direct effect, indirect effect, and total effect after coefficient decomposition respectively. From R^2^ and the Sigma^2^ and log-likelihood statistics, the fit of the model is better and the overall regression reliability is higher. As column (1) shows, the Treated ∗ time coefficient is −0.141, and is significant at the 1% level. This means that the establishment of carbon emissions trading pilot cities can alleviate local urban carbon intensity, which is consistent with the results of the benchmark in front of the regression. Column (2) shows that the Treated ∗ time coefficient is 0.168 and is significant at the 1% level. This means that the establishment of pilot emissions trading can increase the carbon intensity in areas surrounding the region. W ∗ Treated ∗ time before the time coefficient is 0.399 and is significant at the 1% level. This means that, when pilot emissions trading was set up in this region, the ETS produced a spatial spillover effect, increasing the carbon intensity of the surrounding area. Because of the region’s strict carbon trading controls, polluting companies cannot afford the high prices of carbon credits and move to surrounding areas. As the above result shows, the establishment of a pilot emissions trading city not only can reduce the carbon intensity of the city, but also can affect surrounding cities. The environmental regulation in the region, through the strict design of the carbon trading system, relies on the power of the government. The expansion of the implementation of tertiary industries such as service, acceleration of the upgrading of industrial structure, and finally, the improved efficiency of energy utilization can alleviate the carbon intensity of the region, but may cause enterprises to transfer, which can increase the carbon intensity in the surrounding areas. This proves hypothesis 4.

## 7. Conclusions and Recommendations

### 7.1. Conclusions

This paper regards the carbon emission trading system as a quasi-natural experiment. Using the panel data of 281 cities in China from 2006 to 2019, this paper empirically examined the policy effect and spatial spillover effect of ETS on urban carbon intensity in China by using PSM-DID and spatial Durbin models and analyzed it from multiple perspectives.

First, ETS helps mitigate urban carbon intensity. However, this effect has heterogeneous characteristics. The mitigation effect of the carbon emission trading system on the carbon intensity in the eastern region is not significant. By contrast, the mitigation effect on carbon intensity in the central and western regions is very significant. Compared with the central and western regions, the eastern region has a large population, developed economy and various industries. Industrial and residential consumption of electricity and heat energy is huge. Setting up pilot carbon emission trading in the eastern region, while also promoting economic activities in that region, cannot significantly reduce the carbon intensity of cities in a short period of time. In the central and western regions of China, the population is small, and the level of economic development is weak. Setting up carbon emission trading pilots in those regions can effectively reduce the carbon intensity of the regions. The results of the quantile test show that the emission reduction effect of ETS is more obvious for cities with higher carbon intensity, and the marginal effect of emission reduction is larger for cities with higher carbon intensity, so there is more room for emission reduction.

Second, the parallel trend test shows that the longer the carbon emission trading system is established, the more obvious is the mitigation effect on urban carbon intensity. The longer the carbon emission trading system is established, the more time the pilot enterprises have to carry out technological innovation, and the more obvious the effect of mitigating urban carbon intensity measurement will be.

Third, this paper further analyzes the influence mechanism of ETS from the two aspects of green technological innovation and environmental governance. The results show that the carbon emission trading system can encourage enterprises to carry out technological innovation to reduce emissions, thus alleviating urban carbon intensity. By improving the level of environmental governance and reducing the emission of all kinds of pollutants, this also reduces the corresponding carbon emissions, which then alleviates the carbon intensity. This is consistent with most of the literature results.

Fourth, spatial spillovers show that the ETS, although able to mitigate the carbon intensity of the pilot cities, causes the carbon emissions of the surrounding non-pilot cities to rise. This is because the penalty mechanism of ETS leads to high environmental costs that cause enterprises to transfer to surrounding non-pilot areas. As a result, the carbon intensity of surrounding areas increases.

### 7.2. Recommendations

First, the development of ETS should always adhere to the combination of “market determination” and “government regulation”. On the one hand, policy makers should continue to insist on the decisive role of the market in the allocation of carbon emission rights, and should use supply and demand mechanisms, competition mechanisms, price mechanisms, and other means to promote the effective operation of carbon trading markets. This requires constantly adjusting the incentives of enterprises through surplus carbon emission rights and adjusting the penalties imposed on enterprises with insufficient carbon emission rights through market means. Thus, the cost of carbon emissions is internalized into the cost-benefit analysis of the enterprise and becomes an important variable for maximizing corporate profits, thus promoting carbon emission reduction. On the other hand, policy makers should give full play to the regulating and supporting role of the government. The government should formulate laws and regulations suitable for the healthy and effective operation of the market in order to make up for market failures such as monopoly, information asymmetry, and externalities caused by market limitations, thereby constantly improving the market environment.

Second, the carbon reduction effect of ETS is regionally heterogeneous. There are significant differences between different regions due to their local level of economic development, industrial structure, energy structure, and other factors. Therefore, each transaction pilot cannot adopt a “one size fits all” attitude when formulating policies. The construction of carbon trading markets should be carried out according to local conditions. In this way, achieving carbon reduction targets also can promote high-quality economic development.

Third, scientific and technological research and innovation of enterprises is the key to the carbon reduction effect of the ETS. The government, enterprises, and society should pay special attention to the important role of scientific and technological R&D and innovation in carbon trading policies. It is recommended to continuously increase the R&D investment of all enterprises and encourage them to carry out technological innovation in order to constantly update the production process. This will promote the green development of enterprises. The government should also effectively improve its own environmental governance level in order to improve its ability to prevent and control urban pollution. Through the development of a series of laws and regulations to assist the operation of the ETS system, strict penalties should be imposed on enterprises that violate the system.

Fourth, the spatial spillover effect among different cities increases the carbon intensity of surrounding cities. On the one hand, local governments in non-pilot areas are encouraged to actively learn from the experiences of pilot areas in order to reduce the carbon intensity of the region. However, carbon leakage through spatial spillovers undermines the goal of reducing emissions. New mechanisms should be considered to prevent companies from avoiding emission rules. A hybrid mechanism that combines the carbon ETS with other environmental regulation tools is recommended. For example, a carbon tax could be imposed on emissions in non-pilot areas to discourage firms from leaving pilot areas.

The above is the main content of this paper, but the research of this paper still has limitations. This study uses an econometric approach based on historical data from a pilot carbon trading program in China. We also believe that carbon tax is one of the effective ways to reduce the carbon intensity of cities. If carbon tax projects are implemented in these cities, a more reasonable conclusion can be obtained by comparing the effects of carbon trading and carbon tax. Since China has no plan to implement carbon tax at present, such data cannot be obtained to reconstruct the regression model of carbon tax cases. This prevents more reasonable conclusions from being drawn. If China has some concrete practice in carbon tax, the author will study it.

## Figures and Tables

**Table 1:** Description of variables.

Index	Measure
PGDP	Real GDP per capita in cities is measured in logarithms (Yuan per person)
IND	Ratio of the added value of secondary production to the gross city product (%)
PP	The logarithm of the resident population at the end of the year (million)
OPEN	Ratio of foreign investment to gross city product (%)
FS	The ratio of total social loans to gross urban product (%)
FD	The ratio of total social savings to gross urban product (%)
WODK	The proportion of the use of environmental words in the total words of the government work report. (e.g., environmental protection, green, low-carbon, energy-saving and emission reduction, etc.) (%)

**Table 2:** Balance test of propensity score matching.

Variable	Sample Match	The Mean		Standard Deviation (%)		*t* Test *p* > |*t*|
		Treat	Control	Deviation	To Reduce	
PGDP	Before	10.576	10.432	20	99.3	0.000
	After	10.576	10.575	0.1		0.982
IND	Before	3.818	3.844	−11.2	99.3	0.026
	After	3.818	3.836	7.8		0.203
PP	Before	6.084	5.962	19	97	0.000
	After	6.084	6.088	−0.6		0.926
OPEN	Before	0.003	0.003	23.7	95.6	0.000
	After	0.003	0.003	1		0.877
FS	Before	0.725	0.727	−1.2	−550.3	0.824
	After	0.725	0.706	7.8		0.182
FD	Before	0.820	0.812	1.8	−236.8	0.694
	After	0.820	0.794	5.9		0.323
WODK	Before	0.003	0.003	11.5	78.1	0.017
	After	0.003	0.004	-2.5		0.692

**Table 3:** Descriptive statistics.

Variable	Size	Means	Std. Dev.	Min.	Max.
CARBON	3432	−2.876	0.650	−5.113	−0.355
PGDP	3432	10.453	0.691	7.926	13.056
IND	3432	3.840	0.242	2.460	4.450
PP	3432	5.980	0.611	3.959	8.136
OPEN	3432	0.003	0.003	0	0.019
FS	3432	0.727	0.263	0.083	2.547
FD	3432	0.814	0.411	0.112	2.683
WODK	3432	0.003	0.001	0	0.012

**Table 4:** Dual difference regression.

Variable	(1)	(2)	(3)	(4)	(5)
Treated ∗ time	−0.625 ***	−0.173 ***	−0.150 ***	−0.142 ***	−0.316 ***
	(0.264)	(0.026)	(0.024)	(0.024)	(0.027)
PGDP		−0.643 ***	−0.618 ***	−0.636 ***	−0.288 ***
		(0.009)	(0.010)	(0.011)	(0.024)
IND		0.178 ***	0.155 ***	0.248 ***	0.116 **
		(0.034)	(0.031)	(0.038)	(0.051)
PP			−0.616 ***	−0.576 ***	−0.197 ***
			(0.075)	(0.083)	(0.013)
OPEN			1.394	1.759	−3.730
			(1.962)	(1.836)	(2.606)
FS				0.127 ***	0.501 ***
				(0.037)	(0.043)
FD				0.010	−0.109 ***
				(0.034)	(0.027)
WODK				3.211	9.683 *
				(1.96)	(5.746)
Constant	−4.061 ***	1.952 ***	5.448 ***	4.932 ***	−0.911 ***
	(0.001)	(0.156)	(0.427)	(0.488)	(0.298)
Id	YES	YES	YES	YES	NO
Year	YES	YES	YES	YES	YES
R_2_	0.153	0.894	0.903	0.905	0.451
Sample size	3432	3432	3432	3432	2839

Note: *, **, and *** represent the significance levels of 10%, 5%, and 1% respectively. The clustering standard error is shown in brackets.

**Table 5:** Regional heterogeneity test.

Variable	The Eastern Region	The Central Region	In the Western Region
Treated ∗ time	−0.067 *	−0.207 ***	−0.239 ***
	(0.035)	(0.020)	(0.021)
Constant	6.132 ***	4.524 ***	4.777 **
	(1.194)	(0.694)	(1.954)
Control	YES	YES	YES
Id	YES	YES	YES
Year	YES	YES	YES
R_2_	0.909	0.916	0.900
Sample size	1324	1405	703

Note: *, **, and *** represent the significance levels of 10%, 5%, and 1% respectively. The clustering standard error is shown in brackets.

**Table 6:** Quantile regression.

Variable	M = 0.1	M = 0.3	M = 0.5	M = 0.7	M = 0.9
Treated ∗ time	−0.203 ***	−0.220 ***	−0.328 ***	−0.435 ***	−0.520 ***
	(0.055)	(0.033)	(0.021)	(0.028)	(0.045)
Constant	0.049	0.041	0.255	0.459	1.513 ***
	(0.355)	(0.232)	(0.345)	(0.283)	(0.359)
Control	YES	YES	YES	YES	YES
Id	YES	YES	YES	YES	YES
Year	YES	YES	YES	YES	YES
R_2_	0.283	0.286	0.289	0.283	0.264
Sample size	3432	3432	3432	3432	3432

Note: *** represent the significance levels of 1%. The clustering standard error is shown in brackets

**Table 7:** Results of replacing the matched DID.

Variable	Mahalanobis Distance Matches	Caliper Match	Radius of a Match	Nuclear Match
Treated ∗ time	−0.168 ***	−0.141 ***	−0.168 ***	−0.141 ***
	(0.026)	(0.024)	(0.026)	(0.024)
Constant	4.530 ***	4.932 ***	4.530 ***	4.861 ***
	(0.469)	(0.488)	(0.469)	(0.493)
Control	YES	YES	YES	YES
Id	YES	YES	YES	YES
Year	YES	YES	YES	YES
R_2_	0.872	0.905	0.872	0.904
Sample size	3934	3432	3934	3434

Note: *** represent the significance levels of 1%. The clustering standard error is shown in brackets.

**Table 8:** Results of mediating effect test.

Variable	Green Technology Innovation		Environmental Governance	
	(1)	(2)	(3)	(4)
	Inno	Carbon	Trash	Carbon
Treated ∗ time	0.931 ***	−0.055 ***	−0.119 ***	−0.079 ***
	(0.140)	(0.008)	(0.039)	(0.008)
Inno		−0.016 ***		
		(0.001)		
Trash				0.013 ***
				(0.003)
Constant	−30.76 ***	1.884 ***	3.910 **	1.169 ***
	(5.346)	(0.317)	(1.515)	(0.305)
Control	YES	YES	YES	YES
Id	YES	YES	YES	YES
Year	YES	YES	YES	YES
Sobel test	Z = −6.094 ***		Z = −2.333 **	
The Bootstrap test	[−0.023, −0.007] (BC)		[−0.004, −0.0003] (BC)	
R^2^	0.765	0.979	0.851	0.985
Sample size	3432	3432	3432	3432

Note: ** and *** represent the significance levels of 5%, and 1% respectively. The clustering standard error is shown in brackets.

**Table 9:** Results of spatial correlation test.

Year	Moran’s I	Z Value	Year	Moran’s I	Z Value	Year	Moran’s I	Z Value
2006	0.141 ***	27.849	2011	0.128 ***	25.399	2016	0.172 ***	33.931
2007	0.136 ***	26.840	2012	0.128 ***	25.332	2017	0.173 ***	34.062
2008	0.131 ***	25.889	2013	0.133 ***	26.313	2018	0.168 ***	33.182
2009	0.130 ***	25.670	2014	0.143 ***	28.317	2019	0.183 ***	36.064
2010	0.133 ***	26.251	2015	0.156 ***	30.766			

Note: *** represent the significance levels of 1%. The clustering standard error is shown in brackets.

**Table 10:** Regression results of spatial Durbin model.

Variable	(1)	(2)	(3)
Treated ∗ time	−0.141 ***	0.168 ***	−0.141 ***
	(0.024)	(0.026)	(0.024)
W ∗ Treated ∗ time	0.399 *** (0.063)		
Log-likelihood	4258.507		
sigma^2^	0.006 *** (0.001)		
Control	YES		
Id	YES		
Year	YES		
R^2^	0.316		
Sample size	3 934		

Note: *** represent the significance levels of 1%. The clustering standard error is shown in brackets.

## Data Availability

The data in this paper were collected through publicly available data. Data about cities are based on the related data from the National Bureau of Statistics (https://data.stats.gov.cn/, accessed on 15 August 2021). Patent data is from the State Intellectual Property Office (https://www.cnipa.gov.cn/, accessed on 20 August 2021). The relevant data can be downloaded from the relevant website. Data can be obtained from the corresponding author on request.
